# Human lymph node fibroblastic reticular cells maintain heterogeneous characteristics in culture

**DOI:** 10.1016/j.isci.2024.110179

**Published:** 2024-06-04

**Authors:** Janna E.G. Roet, Andrew I. Morrison, Aleksandra M. Mikula, Michael de Kok, Daphne Panocha, Henk P. Roest, Luc J.W. van der Laan, Charlotte M. de Winde, Reina E. Mebius

**Affiliations:** 1 Amsterdam UMC location Vrije Universiteit Amsterdam, Molecular Cell Biology & Immunology, De Boelelaan 1117, Amsterdam, the Netherlands; 2 Amsterdam Institute for Immunology and Infectious diseases, Amsterdam, the Netherlands; 3 Cancer Center Amsterdam, Cancer Biology & Immunology, Amsterdam, the Netherlands; 4 Erasmus MC Transplant Institute, University Medical Center Rotterdam, Department of Surgery, Dr. Molewaterplein 40, 3015 GD Rotterdam, the Netherlands

**Keywords:** Biological sciences, Molecular biology, Cell biology

## Abstract

Fibroblastic reticular cells (FRCs) are mesenchymal stromal cells in human lymph nodes (LNs) playing a pivotal role in adaptive immunity. Several FRC subsets have been identified, yet it remains to be elucidated if their heterogeneity is maintained upon culture. Here, we established a protocol to preserve and culture FRCs from human LNs and characterized their phenotypic profile in fresh LN suspensions and upon culture using multispectral flow cytometry. We found nine FRC subsets in fresh human LNs, independent of donor, of which four persisted in culture throughout several passages. Interestingly, the historically FRC-defining marker podoplanin (PDPN) was not present on all FRC subsets. Therefore, we propose that CD45^neg^CD31^neg^ human FRCs are not restricted by PDPN expression, as we found CD90, BST1, and CD146/MCAM to be more widely expressed. Together, our data provide insight into FRC heterogeneity in human LNs, enabling further investigation into the function of individual FRC subsets.

## Highlights

• Improved isolation and culture of human lymph node-derived FRCs
• Nine heterogeneous clusters of FRCs found in *ex vivo* CD45^neg^ CD31^neg^ cells
• Four out of nine clusters are preserved *in vitro*, while four new clusters emerge
• Donor homogeneity indicates that not all FRCs are PDPN^+^

## Graphical abstract

**Figure:**
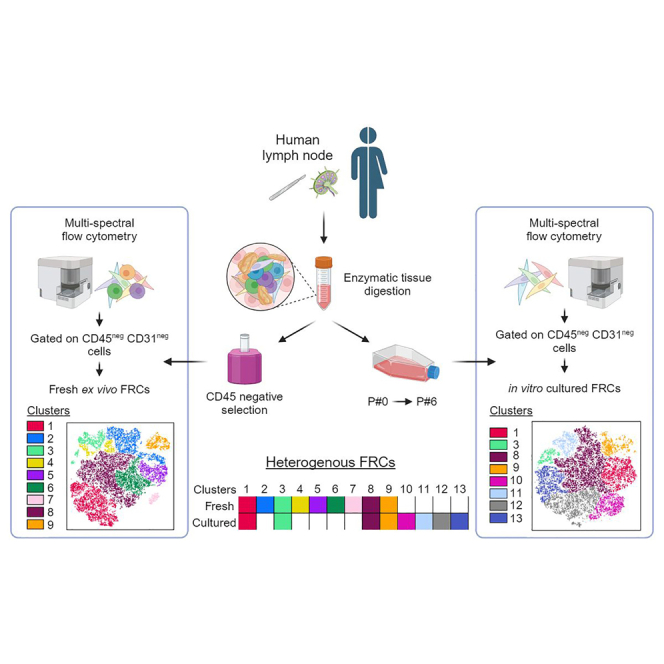

## Introduction

Human lymph nodes (LNs) are strategically positioned throughout the body, allowing cells and antigens from the tissues to enter via lymphatic vessels, while naive lymphocytes enter from the bloodstream by means of specialized endothelial cells forming high endothelial venules (HEVs). Through their lymphatic drainage, LNs function as a filter for tissues, allowing the generation of immune responses upon tissue damage or infection. Within LNs, a high level of organization provides distinct domains for adaptive immune cells, with B cell follicles in the cortex and T cell areas in the paracortex.^1^^,^^2^ Upon presentation of antigen by dendritic cells (DCs) to lymphocytes, the adaptive immune response is initiated. These cellular interactions are influenced by LN stromal cells (LNSCs) that not only provide structural support to LNs but also facilitate cellular migration and anchorage where needed. They also control lymphocyte proliferation, restrict self-reactive T cells, and produce immune cell survival factors.^3^^,^^4^ Thus, non-hematopoietic LNSCs are central to the final outcome of adaptive immune responses.

LNSCs are traditionally subdivided into four main groups using three cell surface markers: CD45, CD31, and podoplanin (PDPN). Cells that express the platelet endothelial cell adhesion molecule CD31/PECAM-1 are from endothelial origin and are either blood endothelial cells (BECs) or lymphatic endothelial cells (LECs), where LECs in addition express the glycoprotein marker PDPN. LNSCs derived from mesenchymal lineage are CD31^neg^ and have been termed either double-negative cells (DNCs) or, when they express PDPN, fibroblastic reticular cells (FRCs). Further subdivision of FRCs based on anatomical location within LNs and phenotypic markers defined T cell zone FRCs (TRCs), B cell zone FRCs (BRCs), pericytes, marginal reticular cells (MRCs), as well as follicular dendritic cells (FDCs).^2^^,^^3^ Single-cell RNA sequencing (scRNA-seq) of both mouse and human LNSCs indicated even more heterogeneity among these stromal cell subsets.^5^^,^^6^^,^^7^^,^^8^^,^^9^^,^^10^^,^^11^ This notion allowed for further definition and identification of their specific roles in regulating the immune response. Distinct functions have been identified for FRC subsets, ranging from the generation of micro-domains for the spatial organization and homeostasis of DC subsets,^9^^,^^12^ providing survival niches for macrophage subsets and plasma B cells,^13^^,^^14^ acting as a conduit system for small molecules,^15^^,^^16^ and forming a cellular interaction niche for B cells, T cells, and DCs at the follicular border.^5^^,^^17^^,^^18^^,^^19^ These functional analyses of FRC subsets have mostly been carried out using cell-specific mutations in mice.

For human FRC subsets, determining these cellular functions requires characterization of stromal cells *in vitro* in two-dimensional (2D) and three-dimensional (3D) systems by means of blocking and directed mutagenesis studies to determine cell-specific functions. Several reports have shown that, in addition, PDPN^neg^ mesenchymal stromal cells with similar characteristics to PDPN^+^ FRCs can be found in human LNs.^20^^,^^21^ While PDPN is used historically as the hallmark FRC marker, this is mostly based on its broad expression on mesenchymal stromal cells within mouse LNs. However, the definition of FRCs has changed over the years, ranging from fibroblasts in the T cell area^22^ to all PDPN-expressing mesenchymal cells in mouse LNs.^23^ A substantial portion of mesenchymal stromal cells within human LNs lacks this expression when freshly isolated.^20^^,^^21^ Therefore, we here aim to further standardize the currently available methods for isolation and culture of LN-derived CD45^neg^CD31^neg^ mesenchymal stromal cells, which we collectively define as FRCs for this study. We assess their phenotypic profile by multispectral flow cytometry to allow the analyses of distinct cellular subsets for future studies.

Hereto, we reproduced and improved the isolation of FRCs from healthy human LN tissue^24^^,^^25^ for *ex vivo* analysis of fresh FRCs and *in vitro-*cultured FRCs. We assessed their phenotypic profile using high-dimensional analysis on cell surface proteins that could potentially distinguish various types of FRCs. Based on pseudotime analysis recently performed on human FRCs,^9^ as well as RNA and protein data from recent publications, we selected the following membrane markers: ACKR4, BST1, CD146, CD21, CD271, CD34, CD90, PDPN, and VCAM1.^5^^,^^6^^,^^9^^,^^20^^,^^21^^,^^26^^,^^27^ We analyzed expression of these markers using multispectral flow cytometry and our in-house developed unbiased approach to correct for the great heterogeneity in autofluorescence (AF) found in both fresh and cultured FRCs.^28^ Upon analysis of CD45^neg^CD31^neg^ cells in fresh human LNs, nine heterogeneous FRC clusters were identified across all human LN donors. Of these, four clusters remained throughout several culture passages, while four new clusters emerged upon FRC expansion *in vitro*. Our findings advance the knowledge of FRC heterogeneity in healthy human LNs and identify which subsets are maintained in *in vitro* cultures. This offers a valuable starting point for their prospective use in functional assays to uncover the role of FRC subsets in human immunity.

## Results

### Reproducible methods for isolation, enrichment, and culture of FRCs from human LNs

**Figure 1:**
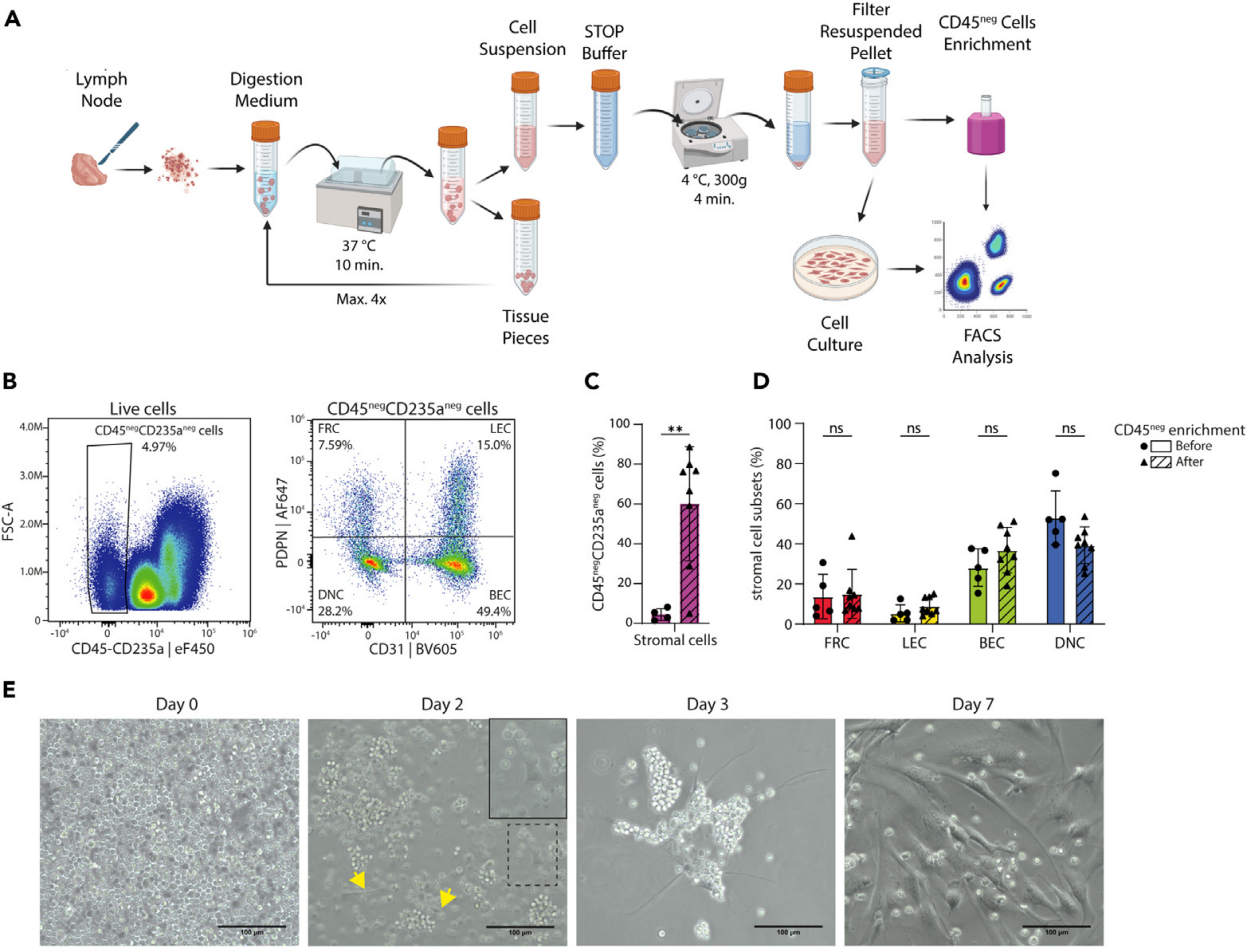
Isolation and CD45^neg^ enrichment for culture of stromal cells from fresh human LNs (A) Schematic representation of the method used to isolate stromal cells from fresh human LNs and the next steps including CD45^neg^ cell enrichment by CD45-negative bead selection or culturing *in vitro*, after which the cells are characterized by spectral flow cytometry (FACS analysis). (B) Representative density plot showing the gating strategy for CD45^neg^CD235a^neg^ cells and subsequently the gating used to determine the four main subtypes of LNSCs based on the expression of PDPN and CD31. (C) Percentage of CD45^neg^CD235a^neg^ stromal cells in the LN cell suspension before (*n* = 4) and after (*n* = 8) CD45^neg^ enrichment. Data represent total mean percentage ±SEM. Unpaired Student’s t test, ∗∗*p* < 0.01. (D) Distribution of the four main LN stromal cell subsets before (*n* = 5) and after (*n* = 8) CD45^neg^ enrichment. Data represent total mean percentage ±SEM. Two-way ANOVA with multiple comparison Tukey’s test, not significant (ns). (E) Representative images of FRC expansion from an LN cell suspension, from day 0, 2, 3, and 7. Scale bar represents 100 μm. See also Figure S1 for cell characteristics of the improved digestion protocol and Table S1 for donor information. FSC, forward scatter; eF, eFluor; BV, Brilliant Violet; FRC, fibroblastic reticular cell; LEC, lymphatic endothelial cell; BEC, blood endothelial cell; DNC, double-negative cell. Created with BioRender.com.

The percentage of FRCs in human LNs is very low (1%–5%) making it a challenge to characterize the phenotypic profiles of the different FRC subsets. Here, we established a protocol to enrich human LNSCs from LN suspensions and to expand FRCs by culturing LN suspensions. To obtain single-cell suspensions from human LNs, we modified the enzymatic digestion protocols published before^24^^,^^25^ (Figure 1A). In short, LNs were minced and subsequently digested with a mixture of three enzymes (Collagenase P, Dispase II, and DNase I). To prevent over-digestion and improve LNSC yield, we performed digestion in 4 cycles of 10 min with gentle shaking of the tube after 5 min, to increase the FRC yield (Figure S1A). After each digestion cycle, the cell suspension was transferred to a buffer containing FCS and EDTA to inhibit enzyme activity and, in contrast to previous protocols, immediately spun down and re-suspended in DMEM-based FRC culture media (in contrast to other medium as mentioned in previous protocols^24^^,^^25^). After digestion, on average, 5% of the cells from the LN cell suspension were identified as CD45^neg^CD235a^neg^ stromal cells (Figures 1B and 1C).

To allow a detailed characterization of these LNSCs, CD45^neg^ stromal cells were enriched to, on average, 60% of the cell suspension using CD45-negative bead selection (Figure 1C), which did not affect PDPN expression (Figure S1B). LNSCs can be divided into four main subtypes, based on the expression of the endothelial cell marker CD31 and the glycoprotein PDPN (Figure 1B).^26^^,^^29^ The CD31-expressing endothelial cells can be divided into LECs (CD31^+^PDPN^+^) and BECs (CD31^+^PDPN^neg^), while mesenchymal subsets, called FRCs, can be divided based on the expression of PDPN into CD31^neg^PDPN^+^ cells and DNCs (CD31^neg^PDPN^neg^). The proportions of these subsets remained the same after CD45^neg^ cell enrichment of the LN cell suspension (Figure 1D).

In order to selectively grow out FRCs, the freshly digested human LN cell suspensions were cultured in collagen type I-coated flasks. After 2–3 days, lymphocytes suspended in the culture flasks were carefully rinsed away. This process of washing away floating cells was consistently repeated twice per week during media refreshments to promote the adherence of cells exhibiting an elongated morphology, i.e., a distinctive characteristic of FRCs (Figure 1E). Using these methods we were able to isolate, culture, and expand FRCs, with most of the cells being attached in a confluent flask at the end of the first culture passage (passage 0) (Figure S1C). There was minimal endothelial stromal cell contamination in passage 2, 4, and 6, as the average percentages of CD31^+^PDPN^neg^ cells were 0.78%, 0.49% and 0.22%, respectively, and the average percentages of CD31^+^PDPN^+^ cells were 0.60%, 0.58%, and 0.28%, respectively (Figure S1D).

### Identification of cell membrane markers to distinguish various subsets of FRCs

**Figure 2:**
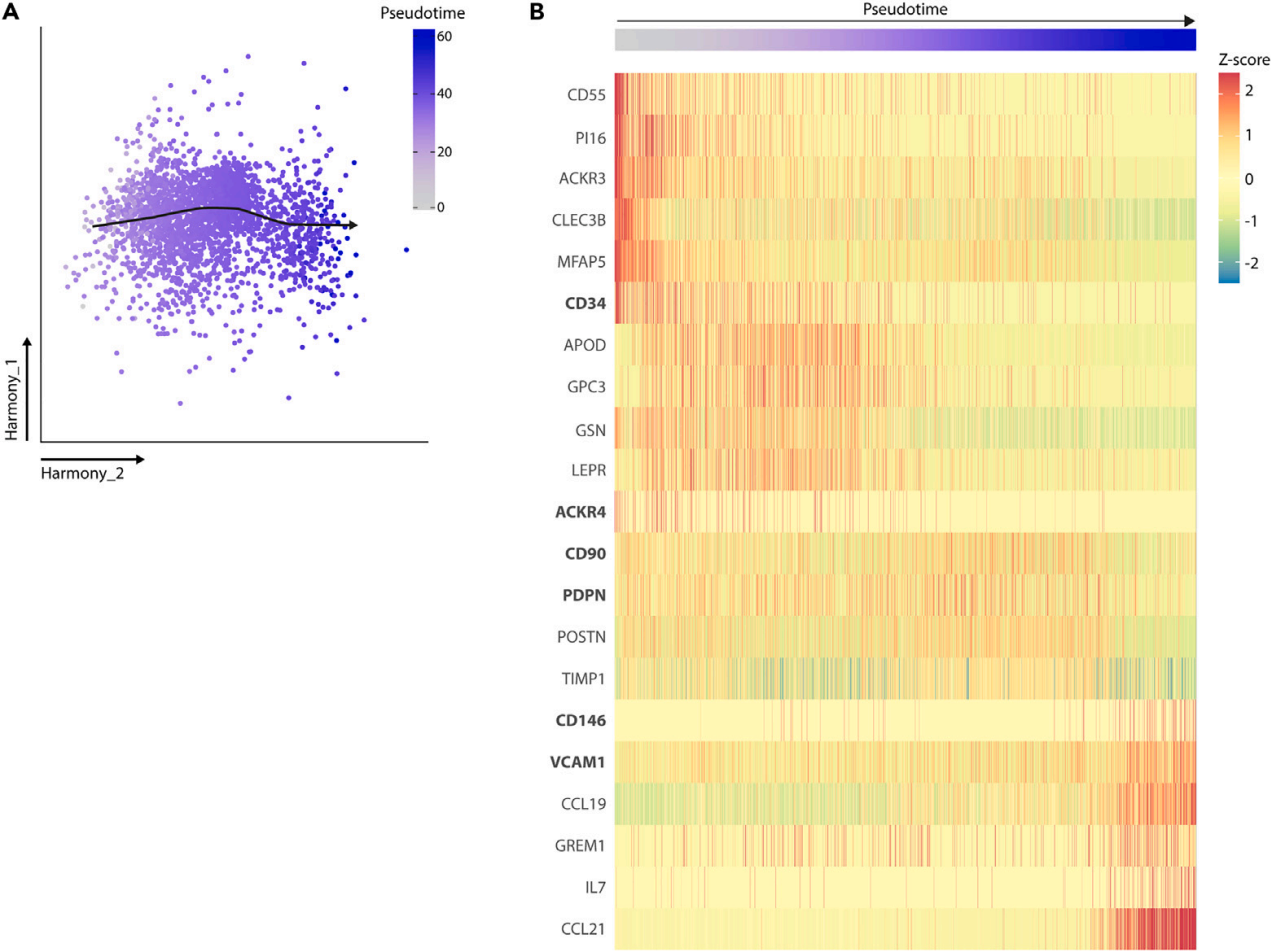
Pseudotime analysis of human LN FRCs (A) Trajectory analysis of human FRCs (CD45^neg^CD31^neg^PDPN^+^) with slingshot obtained from Kapoor.^9^ (B) Heatmap displaying the average expression of indicated genes, including the FRC cell surface markers that we used in this study for flow cytometry analysis, along the pseudotime trajectory as shown before.^9^

Recent studies using scRNA-seq, flow cytometry, and/or imaging have shown that various FRC subsets can be distinguished depending on their marker expression profiles. Based on protein and RNA expression data from mouse LNSCs, PDPN,^26^ BST1,^26^ CD21,^27^ VCAM1,^27^ CD34,^5^ and ACKR4^6^ have all been described as markers for multiple stromal cell subtypes with different localizations and functions within the LN. Additional markers have been described for human LN FRCs, namely CD90,^30^ CD146,^20^ and CD271.^31^ For example, PDPN^+^BST1^+^CD146^+^CD271^+^ cells can be referred to as T-zone FRCs, whereas CD271^+^ cells, along with CD21^+^ and VCAM1^+^ cells, have been used to identify FDCs within the B cell follicles. CD34^+^ stromal cells are located in the LN capsule and medullary vessel adventitia, and ACRK4^+^ stromal cells are located within the subcapsular sinus.^20^^,^^21^ Moreover, re-analyzing the pseudotime analysis of human LN FRCs based on scRNA-seq,^9^ the FRC marker profiles are expressed along the complete pseudotime axis (Figure 2). This suggests that these markers can be used to identify FRC subsets present at different pseudotime stages, and therefore we decided to include them in our analysis.

### Similar FRC subsets are present in freshly isolated human LNs from multiple donors

**Figure 3:**
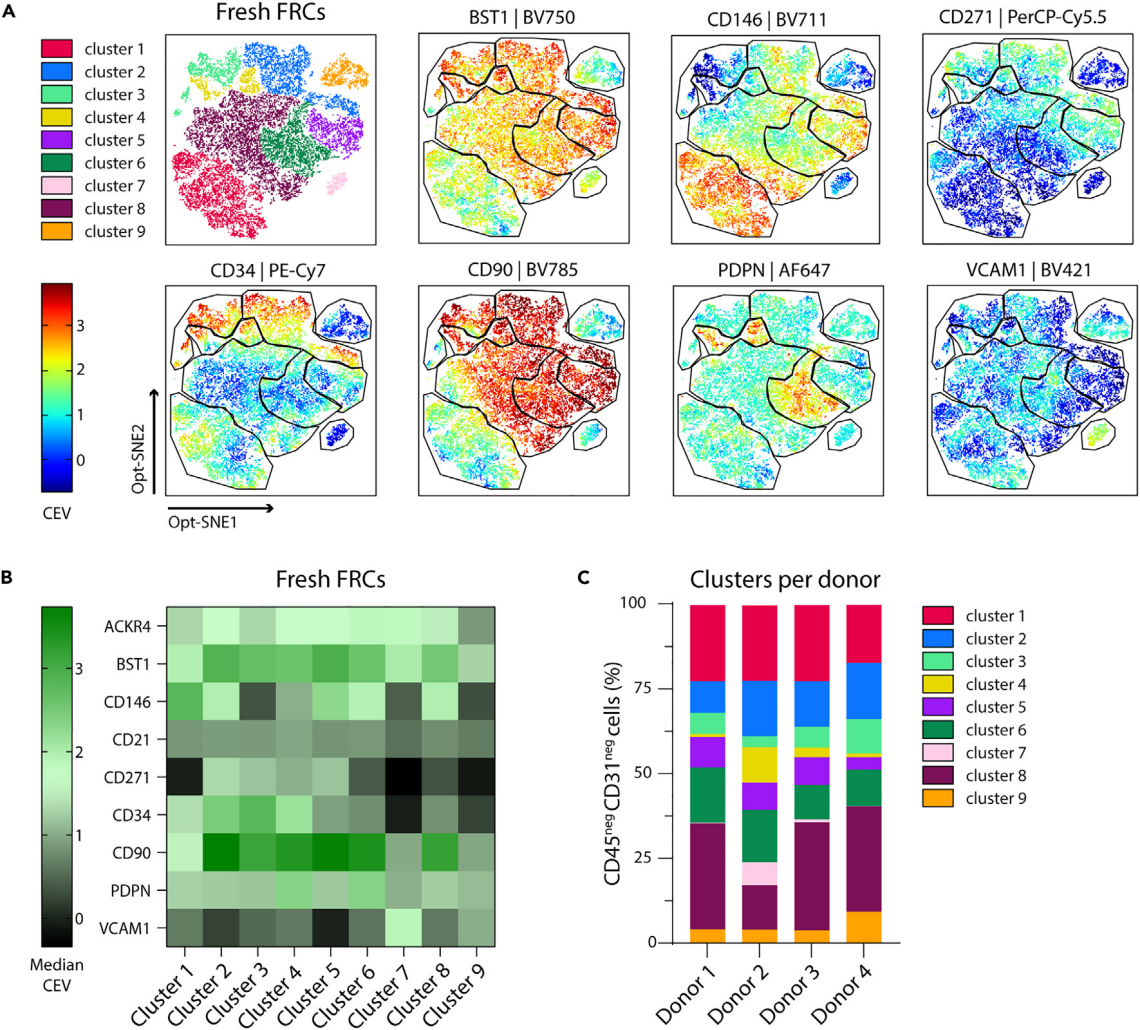
High-dimensional analysis of FRCs in fresh human LN cell suspensions (A) Based on Opt-SNE visualization, we identified nine distinct clusters of fresh FRC subsets, gated from live, CD45^neg^CD31^neg^ cells. Scale bar represents median correction expression value (CEV) from −0.7 to 3.9. Data represent an overlay of four human LN donors (Table S1). (B) Heatmap displaying median CEV of each marker per cluster shown in A. (C) Contribution of clusters (shown in A) to overall CD45^neg^CD31^neg^ cell populations by donor. See also Figure S2 for the gating strategy of the identified clusters, Figure S3 for Opt-SNEs of additional markers and per donor visualization, Table S1 for donor information, and Table S2 for key markers per cluster.

For the characterization of freshly isolated FRCs, we performed multispectral flow cytometry analysis of four human LNs (donors 1–4, Table S1) using the FRC markers described earlier: BST1, CD34, PDPN, CD146, CD271, CD90, VCAM1, CD21, and ACKR4.^5^^,^^6^^,^^20^^,^^21^^,^^26^^,^^27^ AF extraction was performed according to the method developed in our lab,^28^ and we selected live CD45^neg^CD235a^neg^CD31^neg^ cells. To eliminate batch effects, data were corrected according to the integration of multibatch cytometry (iMUBAC) workflow.^32^ Next, dimensional reduction and gating based on the expression of the selected FRC markers (Figure 2) identified nine FRC clusters (Figures 3A, S2, and S3; Table S2), which were all present in the fresh cell suspensions of the four LN donors (Figure S3B).

To better visualize and quantify the median corrected expression value (CEV) of each marker per cluster, a heatmap was generated based on cells from all donors (Figure 3B). The highest median CEV was that of the marker CD90, which was highly expressed across the majority of clusters, apart from low expression in cluster 1, 7, and 9. This pattern of expression was similar to that of BST1, although BST1 CEV was overall lower. Interestingly, expression of PDPN, a historically known FRC-defining marker,^26^^,^^29^ was only expressed in two clusters, 4 and 6. Of these PDPN^+^ subsets, cluster 4 co-expressed BST1, CD90, and CD34, and cluster 6 co-expressed BST1, CD90, and CD146. Additionally, CD34^+^ cells were mostly found in clusters 2, 3, and 4, and CD146^+^ cells were present in clusters 1, 2, 5, 6, and 8. Of note, cluster 7 was the only cluster to show VCAM1^+^ cells with co-expression of BST1 and ACKR4.

We next sought to investigate the contribution of each cluster per donor, and this revealed a consistent percentage of cluster contributions across all donors from freshly isolated CD31^neg^ cell populations (Figure 3C). However, in one donor (donor 2), there was a higher abundance of clusters 4 (CD34^+^PDPN^+^CD90^+^) and 7 (PDPN^neg^CD90^neg^VCAM1^+^) compared to the other donors, and this was compensated with less cells belonging to cluster 3 (CD34^+^PDPN^neg^CD90^+^) and 8 (PDPN^neg^CD90^+^), albeit not statistically significant. Together, based on a selection of nine membrane markers, we identified nine different FRC clusters in fresh cell suspensions of human LNs, and this heterogeneity was consistent over all donors tested.

### FRC subset heterogeneity is maintained in culture throughout different passages

**Figure 4:**
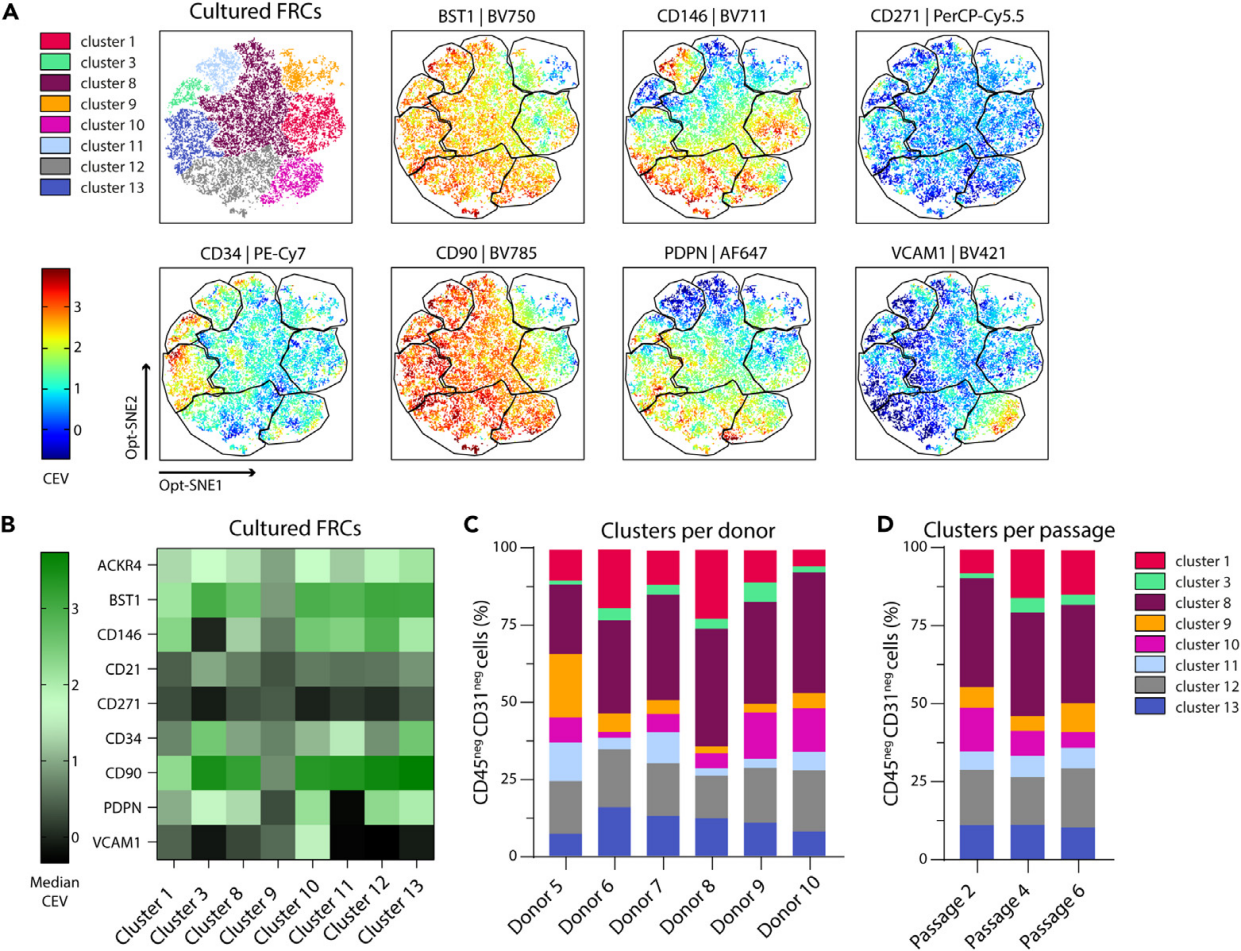
High-dimensional analysis of cultured FRCs (A) Based on Opt-SNE visualization, we identified eight distinct clusters of FRC subsets throughout culture, gated from live, CD45^neg^CD31^neg^ cells. Scale bar represents median correction expression value (CEV) from −0.7 to 3.9. Data represent an overlay of six human LN donors (Table S1), passages 2, 4, and 6 combined. (B) Heatmap displaying median CEV of each marker per cluster shown in A. (C and D) Contribution of clusters to overall CD45^neg^CD31^neg^ cell populations by either (C) donor (mean of all passages combined) or (D) passage number (mean of all six donors combined). See also Figure S2 for gating strategy of the identified clusters, Figure S4 for Opt-SNEs of additional markers, per donor and per passage visualization, Table S1 for donor information, and Table S2 for key markers per cluster.

To investigate if heterogeneity of FRCs is maintained upon *in vitro* culture and over time, we cultured and expanded FRCs from LN cell suspensions from six different donors (donors 5–10, Table S1) and performed multispectral flow cytometry analysis after passage 2, 4, and 6 in the same way as the fresh LN cell suspensions. Using dimensional reduction (Opt-SNE), we identified eight FRC clusters in cultured FRCs (Figures 4A, S2, and S4). Based on the data from marker profiles of fresh FRCs (Figure 3), we observed a conservation of clusters 1, 3, 8, and 9 and a loss of clusters 2, 4, 5, 6, and 7. Interestingly, we identified the emergence of four new clusters: cluster 10 (VCAM1^+^CD90^+^BST1^+^CD146^+^,PDPN^+^), cluster 11 (CD90^+^BST1^+^CD34^+^CD146^+^PDPN^neg^), cluster 12 (CD90^+^BST1^+^CD146^+^PDPN^+^), and cluster 13 (CD90^+^BST1^+^CD34^+^CD146^+^PDPN^+^) (Figure 4A; Table S2).

Next, we generated a heatmap from all donors and combined passages to visualize how the expression of markers deviates per cluster (Figure 4B). The expression of CD90, which was the most prolific marker expressed on FRCs in fresh human LNs (Figure 3), remained highly abundant across most clusters, as well as in the four new clusters (10, 11, 12, and 13) (Figure 4B). This trend was also seen for BST1^+^ and CD146^+^ cells. Furthermore, we noticed the appearance of PDPN^+^ cells in the new clusters 10, 12, and 13 (Figure 4B). In addition, we identified a new VCAM1^+^ cluster (10) co-expressing BST1, PDPN, CD90, and CD146 (Figure 4B), while the VCAM1^+^ FRCs (cluster 7, Figure 3) in *ex vivo* human LNs only co-expressed BST1 and ACKR4.

We next determined the contributions of each cluster per donor across all passages. All donors contributed to all clusters (Figure 4C). However, there was a significant difference between donors for cluster 1 (CD146^+^), 8 (CD90^+^BST1^+^), and 9 (negative for all) (Figures S4B and S4C). Subsequently, we questioned whether all passages contributed uniformly to different clusters. We observed no significant differences in cluster dominance between passages 2, 4, and 6 (Figure 4D). However, cluster 1 (CD146^+^) showed a higher contribution in passage 4 and 6 when compared to passage 2, and this was compensated with more cells belonging to cluster 10 (VCAM1^+^CD90^+^BST1^+^CD146^+^PDPN^+^) in passage 2 (Figure 4D). Together, these data indicate that the heterogeneous FRC phenotype found in human LNs is highly maintained throughout culture irrespective of passage, with a broad overlap of FRC clusters between freshly digested human LNs and cultured cells, and with a consistent homogeneity among all donors.

## Discussion

Here, we provide an updated protocol for isolation and culture of mesenchymal stromal cells from human LNs, which we here collectively call FRCs. This protocol is adapted from previously published protocols for mouse and human LNs^25^ and human tonsils.^24^ Furthermore, we characterized FRC subsets *ex vivo* and at different passages upon culturing and found that FRC heterogeneity is maintained *in vitro*. We observed some differences between FRC clusters *ex vivo* and in culture, as previously reported.^21^ However, changes in expression of markers on LNSC subsets upon culture cannot discriminate whether functionally distinct FRC subsets are lost upon *in vitro* culture or whether they undergo differentiation resulting in expression of additional surface markers.

Based on mouse data, mesenchymal stromal cells can be distinguished from endothelial cells based on lack of CD31 expression.^26^^,^^29^ Within the mesenchymal fraction of murine stromal cells, FRCs are characterized as high PDPN-expressing cells (PDPN^++^), which separate them from a heterogeneous population of CD31^neg^PDPN^neg^ cells (DNCs).^26^^,^^29^ In our data, we find only two PDPN^+^ clusters (out of nine) in the CD31^neg^ fraction in human LNs*.* However, we find five CD90^+^ clusters, of which only two are co-expressing PDPN, suggesting that not all FRCs in human LNs express PDPN, in contrast to mouse LNs. Indeed, Knoblich et al. also found CD90^+^PDPN^neg^ cells in freshly isolated human LNs,^21^ and CD90 has been used as human FRC marker by others.^9^^,^^20^^,^^30^^,^^33^ As such, we conclude that characterization of human CD45^neg^CD31^neg^ FRCs should not be restricted to only PDPN expression as, in addition to CD90, we also found BST1 and CD146/MCAM to be more widely expressed.

However, we also found CD90^neg/dim^ populations that lacked CD146, CD34, PDPN, and BST1, which could indicate that these are the cells originally classified as DNCs.^26^^,^^29^ Recently, CD21 was proposed to further distinguish FRCs from DNCs in the CD31^neg^ fraction of human LN suspensions.^34^ However, in our studies, we did detect some CD21^+^ cells in fresh LN cell suspensions, but they did not form a separate CD21-expressing cluster, neither in fresh LNs nor in cultured FRCs. Likewise, the mesenchymal cell marker CD271, originally detected in splenic FRCs,^31^ was detected at low levels in fresh LNs, but later lost in cultured FRCs.

Using scRNA-seq, nine and twelve mesenchymal stromal cells clusters were defined in mouse LNs^5^^,^^9^ and five in human LNs.^9^ Based on protein expression of key surface markers (BST1, CD146, CD34, CD90, PDPN, and VCAM1), we found nine clusters in fresh human LNs. Of these, four were also present in cultured FRCs, as well as four new clusters (Table S2). We found three CD34^+^ clusters (clusters 2–4) in fresh LNs, which is in line with two *CD34*^*+*^ clusters found by Kapoor et al. using transcriptomics.^9^ According to their pseudotime analysis, CD34-expressing FRCs would form the subset that is placed at the beginning of this trajectory and could thus be the more immature FRC subset present within human LNs.^9^ However, they did not observe co-expression of *CD34* and *CD90* mRNA, whereas in our data all three CD34^+^ clusters co-express CD90 at protein level, which is in line with published flow cytometry data of CD34^+^ LNSCs.^20^ We speculate that *CD34* mRNA is not newly synthesized upon FRC maturation but that CD34 protein expression remains stable. Future research is necessary to test this hypothesis; for example, cellular indexing of transcriptomes and epitopes-sequencing (CITE-Seq) could be used to directly compare scRNA-seq and protein datasets.

We also define a CD90^+^BST1^+^CD146^+^CD271^+^PDPN^neg^ cluster (cluster 5) in human LNs. These cells are most likely pericytes, which are located around blood vasculature controlling endothelial barrier integrity, based on the phenotypic similarity described earlier for human LN pericytes.^20^ Interestingly, we completely lose this cluster upon culture, which indicates that pericytes require other conditions to maintain their characteristic phenotype in culture. Perhaps that co-culture with BECs, which were not present in our cultures (Figure S1D), may allow sufficient cellular interaction needed to mimic and maintain their LN niche.

We found one cluster highly expressing VCAM1 and BST1 (cluster 7) in *ex vivo* FRCs. FDCs, an FRC subset present in B cell follicles, express high VCAM1,^34^ but we did not observe CD21 co-expression in these cells, nor throughout their culture period (Figure S2A). Upon culture, we observed a VCAM1^+^ cluster co-expressing BST1 and PDPN as well as CD90 and CD146. We also detected this FRC phenotype in an organotypic human LN model in which we identified these FRCs as supporting stromal cell type for DCs.^35^ These FRCs also expressed *GREM1* mRNA^35^ and are thus likely to represent the *GREM1*^*+*^ *VCAM1*^*+*^ FRCs that provide a niche maintaining LN-resident DCs.^9^ To more accurately define the other FRC clusters we found in this study, future work needs to elucidate the transcriptome, functionality, and secretome of these cells.

Interestingly, we observed five PDPN^+^ clusters in cultured FRCs compared to two clusters expressing PDPN in *ex vivo* human LNs. Increased PDPN expression is a hallmark of fibroblast activation, for example, described to occur upon wound healing,^36^ during an immune response^37^ or in cancer.^38^ Potentially, the culture medium or growing cells on plastic may induce FRC activation. Furthermore, we expanded and cultured FRCs on collagen type I, which may result in the selective expansion of FRC subsets or induce FRC differentiation. It would be interesting to further investigate phenotypes of FRCs grown on other ECM types, e.g., either single ECM proteins or human LN-derived ECM,^39^^,^^40^ and compare whether these new culturing methods result in the outgrowth of different subsets or the differential contribution of other subsets to the overall stromal cells grown *in vitro*.

Other micro-environmental factors may also influence the observed FRC phenotypes. Here, we have analyzed FRCs from liver-draining LNs, but LNs from other locations in the body, draining other organs, may potentially have different or additional FRC subsets. Additionally, FRCs normally reside in a 3D reticular network, while here they were taken out of this 3D structure for analysis in a single-cell suspension or after 2D culture. We have recently shown that co-culturing FRCs with DCs in a hydrogel induces a DC-supporting phenotype,^35^ which provides a starting point for future investigations of FRC phenotypes in 3D models mimicking the human LN.

Since the markers we used to distinguish these FRC subsets were based on existing datasets, we also took into account the antibody fluorophore availability based on the high AF profile seen in spectral flow cytometry, for which we have developed an analysis pipeline to extract AF spectra.^28^ Spectral flow cytometry allows for analysis of more markers than conventional flow cytometry and can also distinguish various AF signals. However, the number of markers that can be analyzed with spectral flow cytometry is limited by availability of antibodies with correct fluorophores and study of multiple markers on the same cell. The latter depends on the type of cell, density of the marker, chosen fluorophore, and co-expression/co-localization of the marker on the cell surface. When adding additional markers to the antibody panel used in this manuscript, it would be recommended to avoid fluorophores that overlap with channels where high AF is detected (Figure S5).

In summary, this study shows that there are various FRC subsets present in human LNs, of which the majority is preserved upon *in vitro* culture, but culture conditions may also induce functionally relevant new FRC subsets. The heterogeneity of FRC subsets defined here in human LNs and in cultured FRCs provides a better understanding of LNSC subtypes and allows the opportunity of sorting specific subsets for future investigations of FRC functionality in homeostasis, disease, and broader immunological research applications.

### Limitations of the study

This study analyzes the phenotypic profile of freshly isolated *ex vivo* FRCs and *in vitro*-cultured FRCs. The main limitation of this work is the small number of stromal cells from the LN material. In human LNs, there is a low abundance of LNSCs (on average 5% of the total LN cell population) of which around 35% are CD45^neg^CD31^neg^ cells. As such, we could not use the same LN material to compare freshly isolated *ex vivo* FRCs with *in vitro*-cultured FRCs from the same donor. Ideally, to assess whether marker expression is changed upon culture, e.g., gain of PDPN or loss of CD271, FRC subsets should be sorted from fresh LN cell suspensions and their marker profile in culture tracked over time.

All donors for *ex vivo* analysis were female, but for the cultured FRCs we had both female and male donors. We did not observe FRC cluster variation across the different donors, indicating a homogeneity that we would also expect for fresh FRCs between the two sexes.

For the *in vitro*-cultured FRCs, we analyzed passages 2, 4, and 6. We could not analyze earlier passages, as we needed to expand FRCs up to passage 2 in order to have enough cells to perform phenotypic analysis, continue the culture to analyze higher passages, and freeze cells as backup. Moreover, we did not analyze higher passages, since no phenotypic changes between passages 2, 4, and 6 were observed.

## STAR★Methods

### Key resources table

**Table:** 

REAGENT or RESOURCE	SOURCE	IDENTIFIER
**Antibodies**		

anti-ACKR4/CCRL1- BV650	BD Biosciences	Cat# 747804; RRID:AB_2872268
anti-BST1/CD157- BV750	BD Biosciences	Cat# 747147; RRID:AB_2871891
anti-CD146- BV711	BioLegend	Cat# 361032; RRID:AB_2800998
anti-CD21- PE-Dazzle594	BioLegend	Cat# 354922; RRID:AB_2750243
anti-CD235a- eFluor450	Thermo Fisher Scientific	Cat# 48-9987-42; RRID:AB_2574141
anti-CD271- PerCP-Cy5.5	BioLegend	Cat# 345112; RRID:AB_11204075
anti-CD31/PECAM-1- BV605	BioLegend	Cat# 303122; RRID:AB_2562149
anti-CD34- PE-Cy7	BioLegend	Cat# 343516; RRID:AB_1877251
anti-CD45- eFluor450	Thermo Fisher Scientific	Cat# 48-0459-42; RRID:AB_2016677
anti-CD90/Thy1- BV785	BioLegend	Cat# 328142; RRID:AB_2734318
anti-PDPN- Alexa Fluor 647	BioLegend	Cat# 337008; RRID:AB_2162063
anti-VCAM1/CD106- BV421	BioLegend	Cat# 305816; RRID:AB_2832596

**Biological samples**		

Lymph node of donor #1 (female, 83 years old)	Erasmus MC, Rotterdam	N/A
Lymph node of donor #2 (female, 16 years old)	Erasmus MC, Rotterdam	N/A
Lymph node of donor #3 (female, 21 years old)	Erasmus MC, Rotterdam	N/A
Lymph node of donor #4 (female, 53 years old)	Erasmus MC, Rotterdam	N/A
Lymph node of donor #5 (male, 75 years old)	Erasmus MC, Rotterdam	N/A
Lymph node of donor #6 (female, 25 years old)	Erasmus MC, Rotterdam	N/A
Lymph node of donor #7 (male, 16 years old)	Erasmus MC, Rotterdam	N/A
Lymph node of donor #8 (male, 28 years old)	Erasmus MC, Rotterdam	N/A
Lymph node of donor #9 (female, 33 years old)	Erasmus MC, Rotterdam	N/A
Lymph node of donor #10 (male, 53 years old)	Erasmus MC, Rotterdam	N/A

**Chemicals, peptides, and recombinant proteins**		

Belzer UW® Cold Storage Solution	Bridge to Life, University of Wisconsin	
RPMI 1640 Medium	Gibco	Cat# 11875093
Dispase II	Sigma-Aldrich	Cat# 04942078001
Collagenase P	Sigma-Aldrich	Cat# 11213857001
DNAse I	Sigma-Aldrich	Cat# 11284932001
EDTA	Sigma-Aldrich	Cat# 03677
Foetal calf serum	Corning	Cat# 35-079-CV
Dulbecco's Modified Eagle Medium (DMEM), high glucose	Gibco	Cat# 11965092
Penicillin/Streptomycin/Glutamine	Capricorn Scientific	Cat# PS-B
Insulin/Transferrin/Selenium	Gibco	Cat# 41400045
Trypsin	Gibco	Cat# 15400-054
Brilliant Stain Buffer	BD Biosciences	Cat# 563794

**Critical commercial assays**		

LIVE/DEAD™ Fixable Blue Dead Cell Stain Kit	Thermo Fisher Scientific	Cat# L34961
MojoSortTM Human CD45 Nanobeads	BioLegend	Cat# 480029

**Software and algorithms**		

Multibatch Cytometry Data Integration for Optimal Immunophenotyping (iMUBAC)	Ogishi et al.^32^	https://github.com/casanova-lab/iMUBAC
R (version 4.2)	The R Foundation	https://www.rproject.org/
Harmony (version 0.2.1)	Korsunsky et al.^41^	https://github.com/immunogenomics/harmony
OMIQ	Dotmatics	www.omiq.ai
FlowJoTM Software (version 10.10)	BD Biosciences	https://www.flowjo.com
Graphpad Prism 9	GraphPad Software	https://www.graphpad.com
Image J	National Institutes of Health	https://imagej.net
Slingshot	Kapoor et al.^9^ Street et al.^42^	https://github.com/kstreet13/slingshot)
Seurat	Stuart et al.^43^	http://www.satijalab.org/seurat)

### Resources availability

#### Lead contact

Further information and requests for resources and reagents should be directed to and will be fulfilled by the lead contact: Prof. Dr. Reina E. Mebius (r.mebius@amsterdamumc.nl).

#### Materials availability

This study did not generate new unique reagents.

#### Data and code availability

All data reported in this paper will be shared by the lead contacts upon request. This paper does not report original code. Any additional information required to reanalyse the data reported in this paper is available from the lead contacts upon request.

### Experimental model and study participant details

#### Tissue collection

Human LNs were obtained from donors and patients from both sexes during liver transplant procedures performed at the Erasmus MC, Rotterdam, The Netherlands, in accordance to the Medical Ethical Committee (Medisch Ethische Toetsings Commissie; METC) of Erasmus MC (MEC-2014-060). All patients (liver transplant recipients) gave written informed consent to use their donor tissue. The LNs were resected along the hepatic artery and portal vein in the porta hepatis from donor livers and diseased patient explant livers. Donor age and sex are shown in the key resources table, and extended donor characteristics can be found in Table S1. Further information regarding donor ethnicity, race, ancestry and socioeconomic has not been documented upon collection of the tissues. LNs were transported in Belzer University of Wisconsin (UW) cold storage solution (Bridge to Life Ltd., London, England, UK) and processed within 72 hours of surgery.

#### Cell culture

To allow for an efficient and selective outgrowth of FRCs from LN cell suspensions, a seeding density of 1.25 x 10^6^ cell suspension per cm^2^ was used on culture flasks that were coated with 2 μg/cm^2^ collagen from calf skin (Sigma-Aldrich). Culture media comprised of DMEM with 10% FCS, 2% Penicillin/Streptomycin/Glutamine and 1% Insulin/Transferrin/Selenium. After three days, lymphocytes were washed away with PBS to allow for optimal FRC growth. This process of washing away floating cells from the same flask was repeated twice per week during medium refreshments. Upon confluence, cells were harvest with PBS supplemented with 0.05% trypsin and 5 mM EDTA for up to 5 minutes maximum. Trypsin was neutralised using culture medium (as above), and FRCs were passaged or collected for flow cytometry analysis. FRCs were used up to and including passage 6 for all individual experiments.

### Method details

#### Enzymatic digestion of human lymph nodes

Human LNs were enzymatically digested, where modifications were made to the time of each digestion cycle (4 x 10 minute intervals) compared to earlier protocols.^24^^,^^25^ In short, LNs were minced and subsequently digested with an enzyme mixture containing RPMI-1640 medium with 2.4 mg/ml Dispase II, 0.6 mg/ml Collagenase P and 0.3 mg/ml DNase I (all from Sigma-Aldrich, St. Louis, MO, USA). To prevent over-digestion and neutralise the digestion enzymes after each cycle, isolated cells were collected in ice-cold phosphate-buffered saline (PBS) supplemented with 2% foetal calf serum (FCS) and 5 mM EDTA, and spun down at 300 g for 4 minutes at 4°C. The cell pellet was re-suspended in 1 ml Dulbecco's Modified Eagle Medium (DMEM) (Gibco, Grand Island, NY, USA) supplemented with 10% FCS, 2% Penicillin/Streptomycin/Glutamine and 1% Insulin/Transferrin/Selenium (Gibco). After the last digestion cycle, all collected cells were filtered through a 100 μM filter and counted. The obtained human LN cell suspension was either cryopreserved, enriched for CD45^neg^ cells or cultured to grow out FRCs.

#### Enrichment of CD45^neg^ cells

LN suspensions were enriched for CD45^neg^ (stromal) cells by negative selection using MojoSort™ Human CD45 Nanobeads (BioLegend, San Diego, CA, USA). The manufacturer’s protocol was followed, with the addition of a fluorescently-labelled antibody against CD45 during the CD45 Nanobeads incubation step to enhance the CD45 signal for flow cytometry. The CD45^neg^ enriched LN cell suspension was further processed for characterisation by flow cytometry.

#### Multispectral flow cytometry

Cell suspensions were stained in a 96-well U bottom plate at 4°C for multispectral flow cytometry analysis. Cells were firstly washed with PBS and stained with a fixable viability dye (LIVE/DEAD™ Fixable Blue Dead Cell Stain Kit, 1:1000, cat. no. L34961, Invitrogen, Paisley, Scotland, UK) for 10 minutes at 4°C. Next, cells were washed with PBS containing either 0.1 % bovine serum albumin (BSA) or 2% FCS (referred to as FACS buffer) prior to Fc-receptor blocking using 10 % normal human serum in FACS buffer, mixed 1:1 with Brilliant Stain Buffer (BSB) (563794, BD Biosciences, Franklin Lakes, NJ, USA). Cells were then incubated with directly-labelled antibodies diluted in blocking buffer and BSB in the following dilutions: anti-ACKR4/CCRL1- 1:100, anti-BST1/CD157-1:200, anti-CD146-1:100, anti-CD21-1:50, anti-CD235a-1:10, anti-CD271-1:100, anti-CD31/PECAM-1-1:100, anti-CD34-1:100, anti-CD45-1:100, anti-CD90/Thy1-1:100, anti-PDPN-1:50, anti-VCAM1/CD106-1:200. After staining, cells were washed two times with FACS buffer and fixed with 2% PFA for 15 minutes at 4°C in the dark, and washed two times with FACS buffer. Samples, single stains on beads and for some markers (PDPN, BST1 and CD90) single stains on cells as well as Fluorescence Minus One (FMO) controls were acquired on Aurora 5-laser Flow Cytometer (Cytek, Amsterdam, The Netherlands). For highly AF samples, area scaling factor was adjusted across all lasers.

#### Multispectral flow cytometry data analysis

First, the heterogeneous and bright AF spectra seen in LNSCs were extracted according to our in-house developed analysis pipeline.^28^ In short, all different AF clusters of the unstained samples were identified in an unbiased method, and their unique AF spectra were used during the unmixing to extract the AF from the full stained samples. This workflow was performed for every new batch acquired.

To test reproducibility of our FRC isolation protocol, LNSCs were derived from ten human LN donors and used in the different experiments (Table S1). For objective inter-batch comparison, data were corrected using the iMUBAC method.^32^ All files were pre-processed by gating on the cells of interest*, i.e.* live CD45^neg^CD235a^neg^CD31^neg^ cells. Doublet exclusion was omitted due to morphology of human LNSCs affecting the FSC, based on brightfield images acquired on CytPix Attune Flow Cytometer (Thermofisher, Waltham, MA, USA) (Figure S6).

Next, batch correction was performed for all files combined, both fresh and cultured LNSCs, as we assumed that minimal biological variability would be present between the samples. The expression values for all markers were batch-corrected with Harmony (version 0.2.1),^41^ as part of the iMUBAC pipeline^32^ in R (version 4.2), using the same parameters as their example script and using each marker directly as an input for batch correction, resulting in the corrected expression values (CEV) per marker. MaxN was chosen based on the minimum number of available cells per batch. The effect of batch correction was visualised using uniform manifold approximation and projection (UMAP) (Figures S7A–S7C). Using the online analysis software OMIQ (www.omiq.ai, Boston, MA, USA), dimensional reduction (Opt-SNE) was performed separately on the batch-corrected freshly digested LNSCs or batch-corrected cultured FRCs. Different clusters were manually gated based on the expression of different markers.

Data were visualised using OMIQ for the Opt-SNEs, FlowJot™ Software (v10.7, TreeStar, Ashland, OR, USA) for density dot plots, and Graphpad Prism 9 software (GraphPad Software Inc., San Diego, CA, USA) for bar graphs and heatmaps.

#### Brightfield imaging

Human LN cell suspension cultures were assessed for selective outgrowth of FRCs identified on morphological changes. Brightfield images were acquired on consecutive days using a microscope (Zeiss AX10, Jena, Germany) equipped with a high-resolution camera (Canon EOS 100D, Tokyo, Japan). The images were processed with ImageJ software.

#### Pseudotime analysis

To identify the position of the FRC markers on the pseudotime axis of human FRC (CD45^neg^CD31^neg^PDPN^+^) scRNAseq dataset^9^ (E-MTAB-10206), we reconstructed the trajectory using the Slingshot algorithm as described by.^9^^,^^42^ First, the Harmony algorithm was used for batch correction between three donors. Next, the Slingshot algorithm was run on the corrected dimensional reduction from Harmony, resulting in a pseudotime score per cell. For visualisation in a heatmap, the cells were ordered based on their pseudotime scores. Markers for each cluster were calculated using the FindAllMarkers function in Seurat^43^ with default parameters and visualised in a heatmap together with the FRC cell surface markers that could be used for flow cytometry analysis (Table S2) and that were present in the dataset.

### Quantification and statistical analysis

#### Statistics

Statistical analysis was performed using unpaired student’s T-test or two-way ANOVA followed by Tukey’s multiple comparison test in GraphPad Prism 9 software (GraphPad Software Inc., San Diego, CA, USA).∗p < 0.05, ∗∗p < 0.01.
