# Supplementary material for: Human lymph node fibroblastic reticular cells maintain heterogeneous characteristics in culture
Source: iScience. 2024 Jun 4;27(7):110179. doi: 10.1016/j.isci.2024.110179 (PMC11233964; doi:10.1016/j.isci.2024.110179)
Supplement: Document S1. Figures S1–S7 and Tables S1 and S2 [file mmc1.pdf]

iScience, Volume *27*

Supplemental information

Human lymph node fibroblastic reticular cells maintain heterogeneous characteristics in culture

Janna E.G. Roet, Andrew I. Morrison, Aleksandra M. Mikula, Michael de Kok, Daphne Panocha, Henk P. Roest, Luc J.W. van der Laan, Charlotte M. de Winde, and Reina E. Mebius

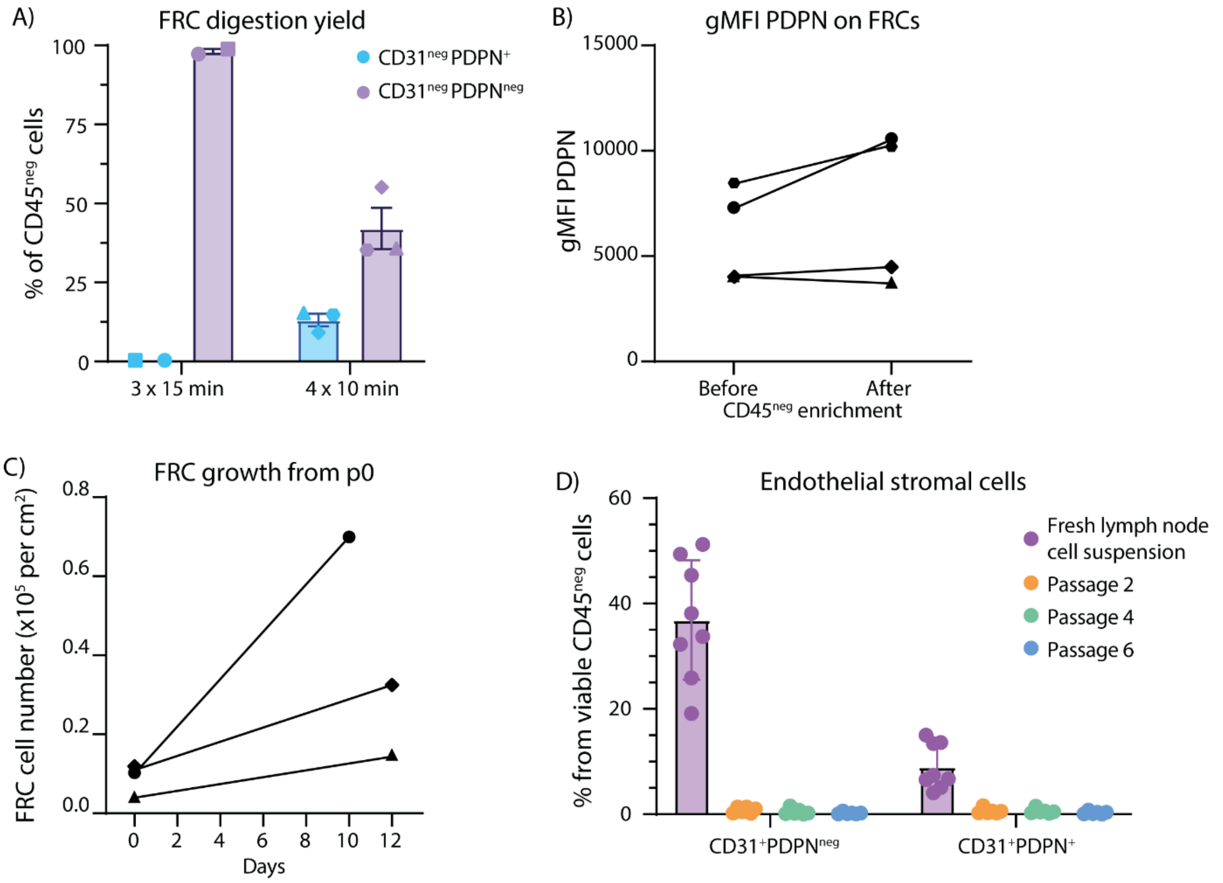

Figure S1. **Cell characteristics of improved digestion protocol, related to Figure 1. A)** Bar graph visualising the percentage of CD31^{neg}PDPN^{+} and CD31^{neg}PDPN^{neg} cells from CD45^{neg} cells after 3 digestion rounds of 15 minutes or after 4 digestion rounds of 10 minutes. **B)** Geometric mean fluorescent intensity of PDPN on FRCs before or after CD45^{neg} enrichment. Shapes represent four different donors. **C)** FRC growth from passage 0, visualised as FRC cell number per cm^{2} on day 0 within the lymph node cell suspensions and after 10 or 12 days in culture. The three different symbols correspond to three different donors. **D)** Bar graph visualising the percentage of endothelial stromal cells from viable CD45^{neg} cells within the fresh lymph node cell suspensions or after culture at passage 2, 4 and 6. For panels A and D, the mean is visualised with error bars representing standard deviations.

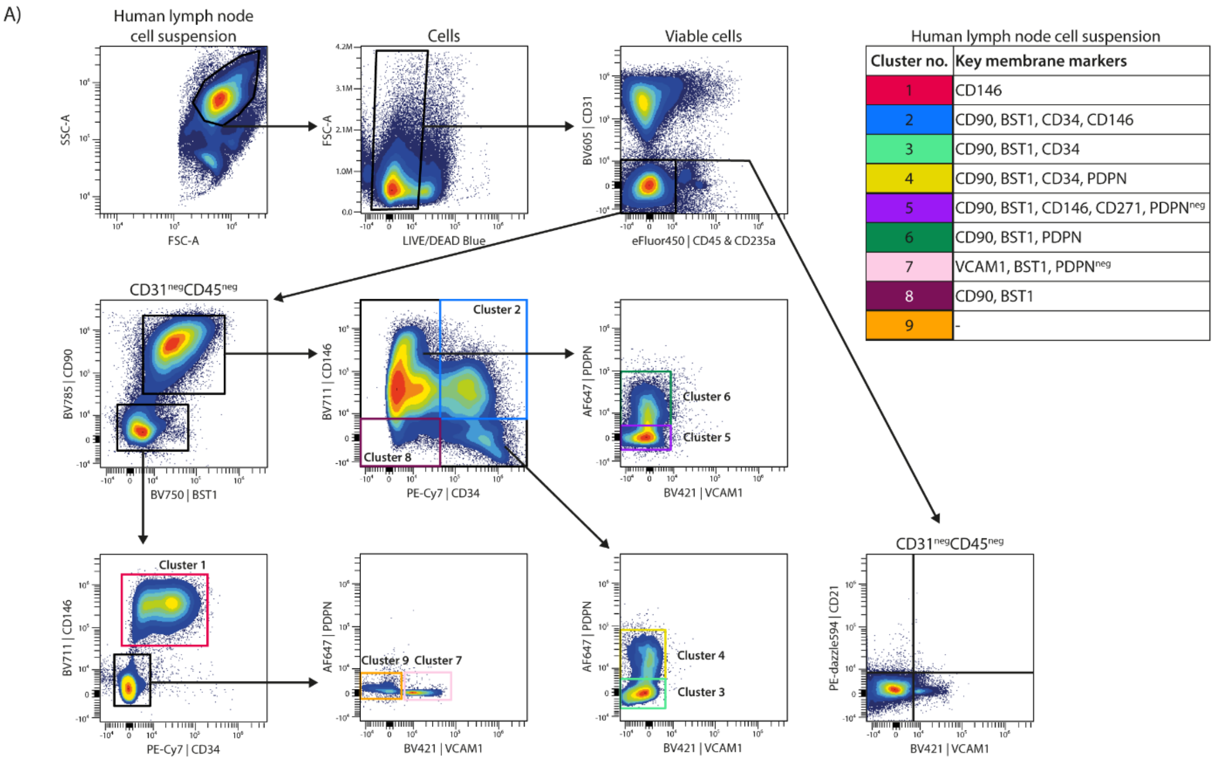

Human lymph node cell suspension

Cluster no.	Key membrane markers
1	CD146
2	CD90, BST1, CD34, CD146
3	CD90, BST1, CD34
4	CD90, BST1, CD34, PDPN
5	CD90, BST1, CD146, CD271, PDPN^{neg}
6	CD90, BST1, PDPN
7	VCAM1, BST1, PDPN^{neg}
8	CD90, BST1
9	-

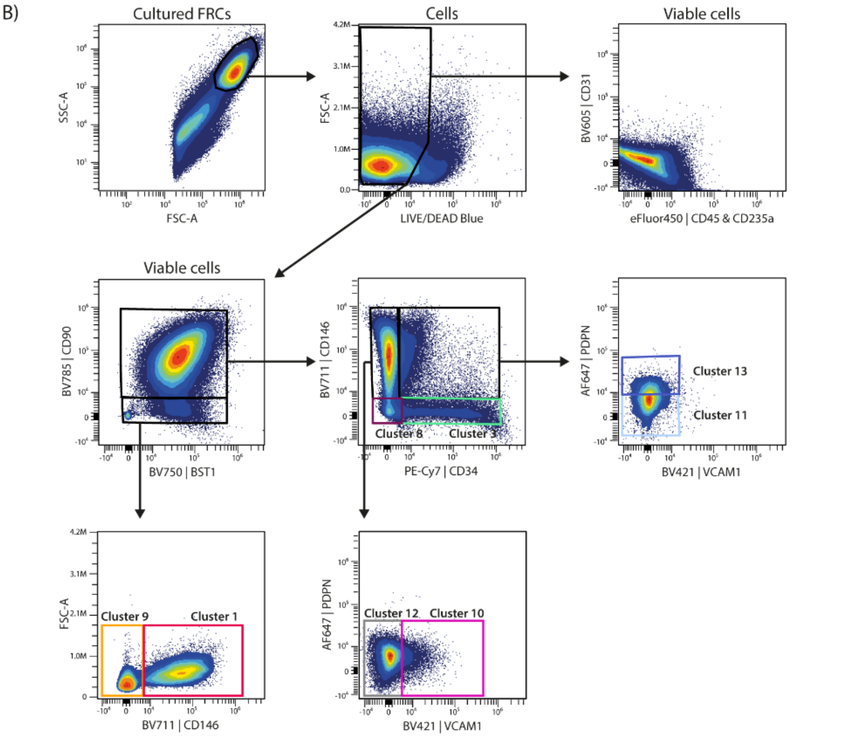

Cultured FRCs

Cluster no.	Key membrane markers
1	CD146
3	CD90, BST1, CD34
8	CD90, BST1
9	-
10	VCAM1, CD90, BST1, CD146, PDPN
11	CD90, BST1, CD34, CD146, PDPN^{neg}
12	CD90, BST1, CD146, PDPN
13	CD90, BST1, CD34, CD146, PDPN

Figure S2. Gating strategy of clusters within human LN cell suspensions and cultured FRCs, related to Figures 3 & 4. A) Gating strategy and key membrane markers of the clusters identified within CD31^{neg}CD45^{neg} cells from human LN cell suspensions, related to Figure 3. Contour plots shown from one representative donor. **B)** Gating strategy and key membrane markers of the clusters identified within CD31^{neg}CD45^{neg} cells from cultured FRCs, related to Figure 4. Contour plots shown from one representative donor.

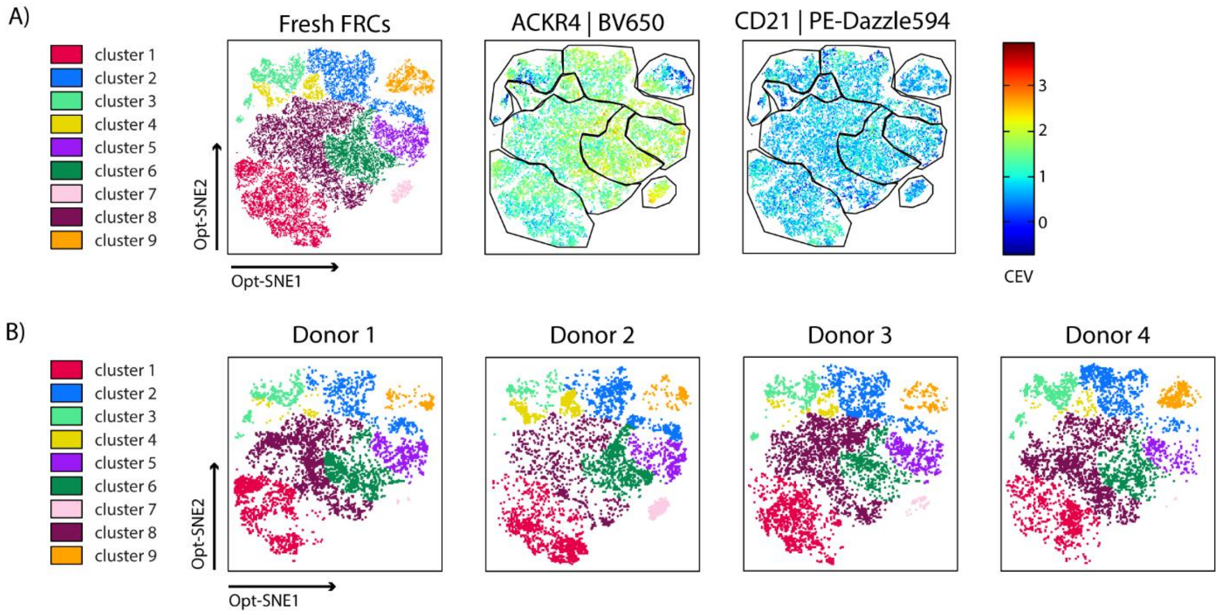

Figure S3. High dimensional analysis of FRCs in fresh human LN cell suspensions, related to Figure 3. A) Based on Opt-SNE visualisation, we identified nine distinct clusters of fresh LNSC subsets, gated from live, CD45^{neg}CD31^{neg} cells. Scale bar represents median correction expression value (CEV) from -0.7 to 3.9. Data represents an overlay of four independent human LN donors (Table S1). **B)** Opt-SNE per human LN donor of the nine distinct clusters of fresh LNSC subsets.

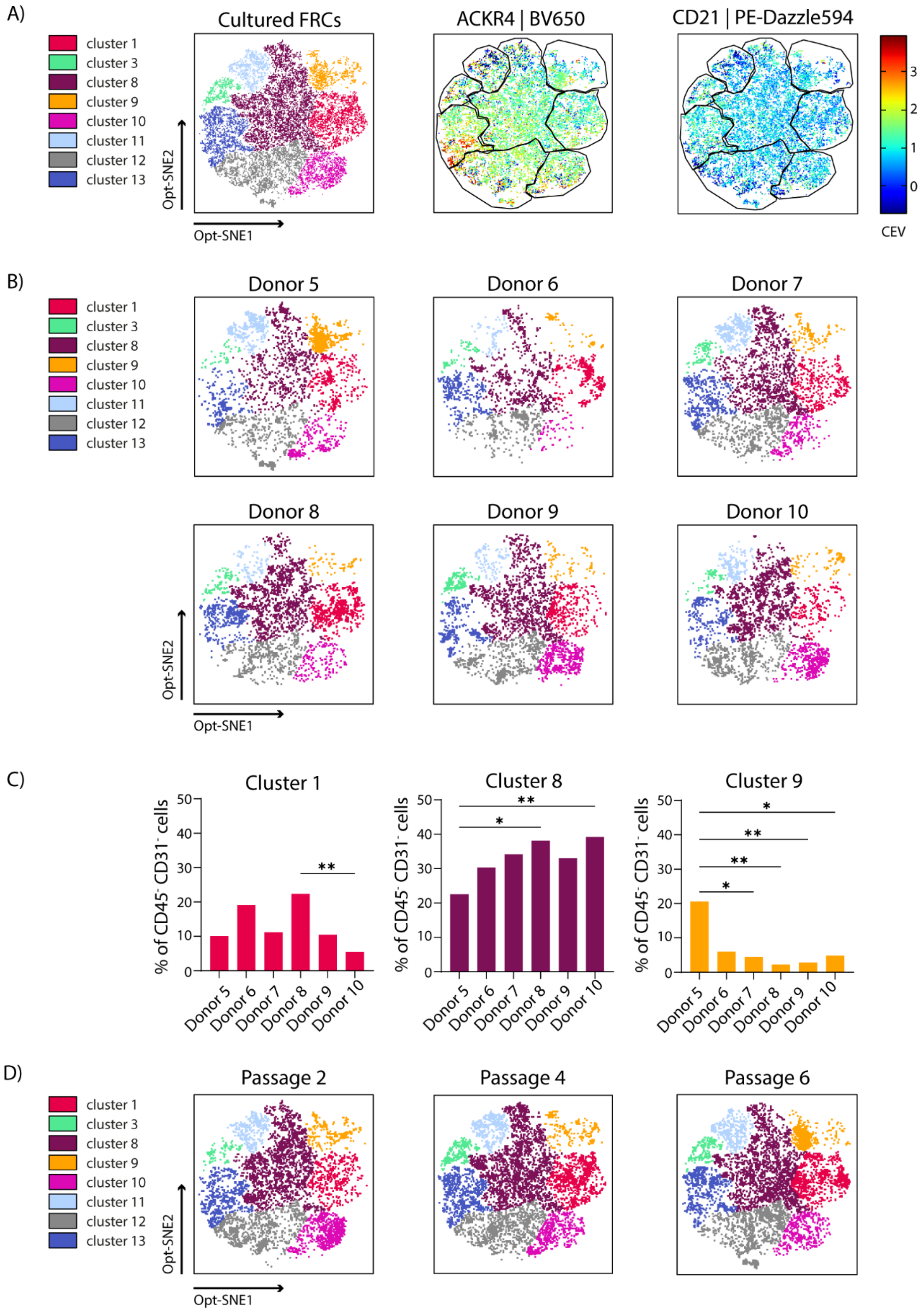

Figure S4. High dimensional analysis of cultured FRCs, related to Figure 4. A) Based on Opt-SNE visualisation, we identified eight distinct clusters of FRC subsets throughout culture, gated from live, CD45^{neg}CD31^{neg} cells. Scale bar represents median correction expression value (CEV) from -0.7 to 3.9. Data represents an overlay of six human LN donors (Table S1), passages 2, 4 and 6 combined. **B)** Opt-SNE per human LN donor of the eight distinct clusters of cultured FRCs, passages 2, 4 and 6 combined. **C)** Significant different clusters between donors are represented per cluster. Data is visualised as mean of three passages per donor. Two-way ANOVA with Tukey's multiple comparison test, $*p < 0.05$ and $**p < 0.01$. **D)** Opt-SNE per passage of the eight distinct clusters of cultured FRCs, six human LN donors combined.

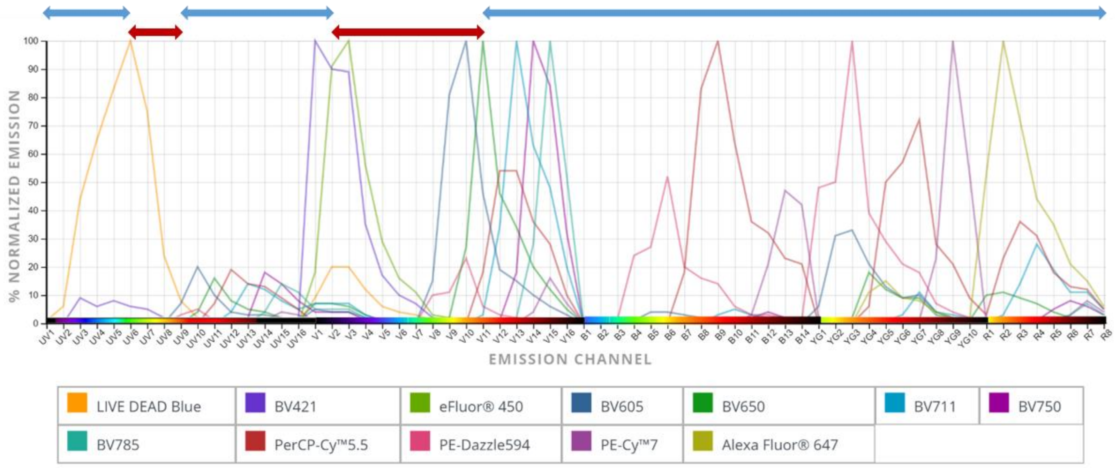

Figure S5. Spectra of the fluorophores of the antibody panel, related to STAR Methods. This image shows the spectra of fluorophores of the antibody panel used in this manuscript (made with Cytek® Spectrum Viewer). The red arrows above the graph indicate areas in the spectrum with high autofluorescence of human FRCs, and the blue arrows indicate areas in the spectrum with low autofluorescence of human FRCs [S1]. When adding extra markers to the panel, we recommend to choose fluorophores emitted in channels with low autofluorescence (blue arrows).

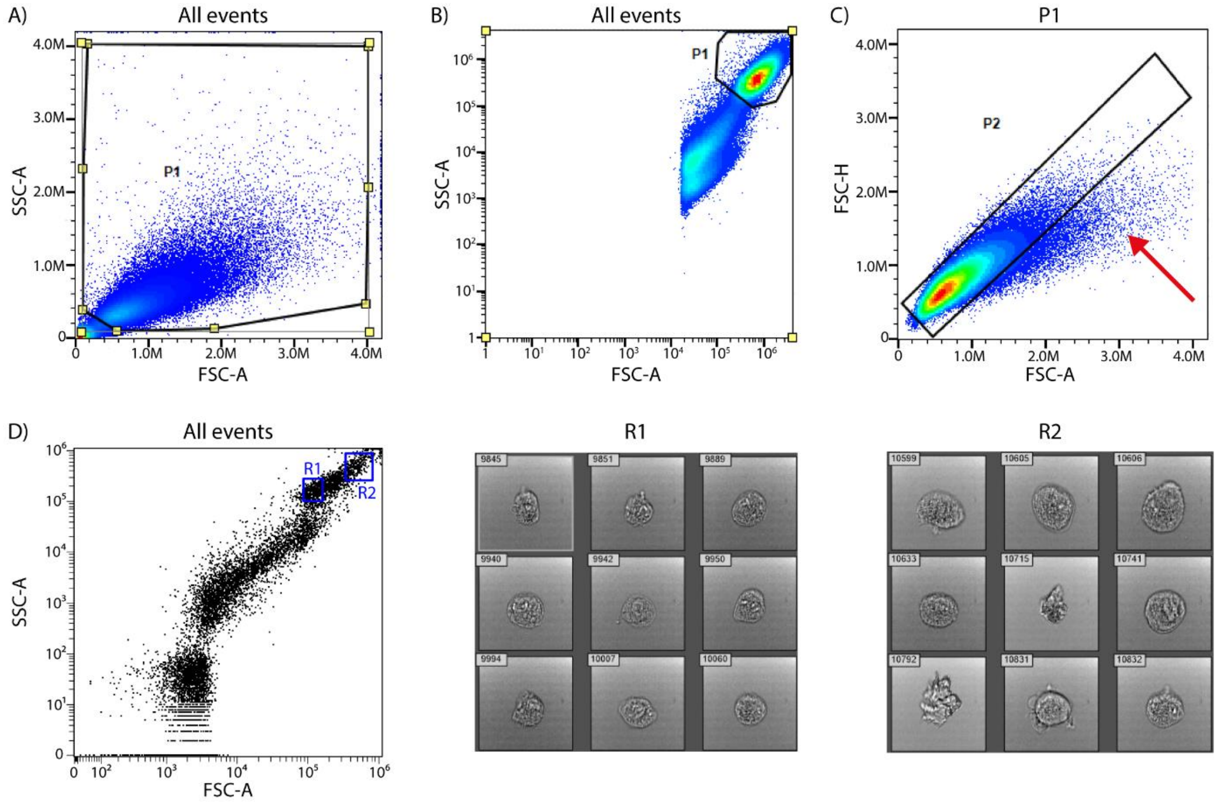

Figure S6. Morphology of cultured human FRCs affects the positioning of events on forward (FSC) and side (SSC) scatter, related to STAR Methods. Gating strategy for selection of human LN stromal cells (gate P1) in **A)** linear and **B)** logarithmic scale. **C)** Red arrow shows single cells deviating from linearity. **D)** Images of individual human FRCs acquired on CytPix flow cytometer. The blue squares indicate the area of zoom in of single events depicted by brightfield images on the right. FSC and SSC are in log scale.

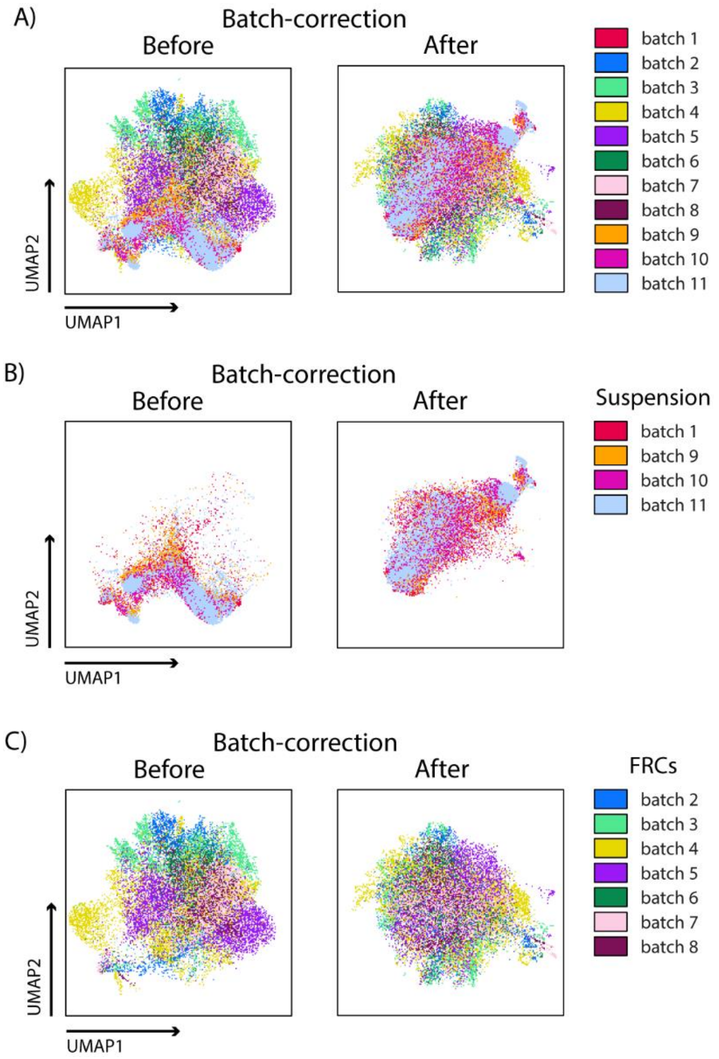

Figure S7. Visualisation of batch correction, related to STAR Methods. A) UMAP visualisation of the different batches before and after iMUBAC batch correction. **B)** UMAP visualisation of the batches of fresh LN cell suspensions before and after iMUBAC batch correction. **C)** UMAP visualisation of the batches of cultured FRCs before and after iMUBAC batch correction.

Table S1. Human lymph node donor characteristics, related to Figures 1, 3 & 4. F: Female, M: Male, DBD: Donation after Brain Death, DCD: Donation after Circulatory Death, DLT: Donation after Liver Transplantation.

#	Sex	Age	Donor type	Underlying liver disease	Used in Figure
1	F	83	DBD	None	1 & 3
2	F	16	DBD	None	1 & 3
3	F	21	DBD	None	1 & 3
4	F	53	DBD	None	1 & 3
5	M	75	DCD	None	4
6	F	25	DLT	Wilson's disease	4
7	M	16	DCD	None	4
8	M	28	DCD	None	4
9	F	33	DCD	None	4
10	M	53	DBD	None	4

Table S2. Summary of FRC subsets present in *ex vivo* and cultured human LNSC samples, related to Figures 3 & 4.

Cluster no.	Present ex vivo?	Present in culture?	Key membrane markers
1	Yes	Yes	CD146
2	Yes	-	CD90, BST1, CD34, CD146
3	Yes	Yes	CD90, BST1, CD34
4	Yes	-	CD90, BST1, CD34, PDPN
5	Yes	-	CD90, BST1, CD146, CD271, $PDPN^{neg}$
6	Yes	-	CD90, BST1, PDPN
7	Yes	-	VCAM1, BST1, $PDPN^{neg}$
8	Yes	Yes	CD90, BST1
9	Yes	Yes	-
10	-	Yes	VCAM1, CD90, BST1, CD146, PDPN
11	-	Yes	CD90, BST1, CD34, CD146, $PDPN^{neg}$
12	-	Yes	CD90, BST1, CD146, PDPN
13	-	Yes	CD90, BST1, CD34, CD146, PDPN

References

[S1] Roet, J.E.G., Mikula, A.M., Kok, M.d., Chadick, C.H., Vallejo, J.J.G., Roest, H.P., Laan, L.J.W.v.d., Winde, C.M.d., and Mebius, R.E. (2023). Unbiased method for spectral analysis of cells with great diversity of autofluorescence spectra. bioRxiv, 2023.2007.2028.550943. https://doi.org/10.1101/2023.07.28.550943.
